# Investigation of transverse crack spacing in an asphalt pavement with a semi-rigid base

**DOI:** 10.1038/s41598-022-23122-y

**Published:** 2022-10-27

**Authors:** Jiangcai Chen, Hengzhen Li, Zihong Zhao, Xiangchen Hou, Junhui Luo, Cheng Xie, Haobin Liu, Tianzeng Ren, Xiaofeng Huang

**Affiliations:** 1 Guangxi Beitou Traffic Maintenance Technology Group Co., Ltd., Nanning, 530201 China; 2 School of Transportation Science and Engineering, Harbin Institute of Technology, Harbin, 150090 China; 3 Guangxi Communications Design Group Co., Ltd., Nanning, 530201 China

**Keywords:** Civil engineering, Scaling laws

## Abstract

Transverse cracking is a serious problem for semi-rigid base asphalt pavement. The shrinkage of the base course and the surface course, as well as reflective cracks, are key factors for transverse cracking in asphalt pavement. Crack spacing can directly reflect the degree of transverse cracking in pavements. Therefore, this study aims to calculate the transverse crack spacing and discuss its affecting factors. To this end, a calculation model of transverse crack spacing for the semi-rigid base asphalt pavement was first established. Then, the transverse crack spacings of different composite structures were calculated, and the influences of the shrinkage coefficient, the structural layer thickness, and the pavement tensile strength on transverse crack spacing were expounded. Finally, the transverse crack spacing of the pavement after the appearance of the reflective was calculated. The results show that the lower lime and fly ash content and skeleton gap gradation can be adopted during the design of the base course. Meanwhile, the lower lime and fly ash content in the macadam base, the skeleton gap gradation and asphalt concrete with a larger particle size in the surface layer can be used during the design of surface layer. In addition, the transverse crack spacing of the semi-rigid base asphalt pavement could be increased by reducing the shrinkage coefficient, increasing the thicknesses of the surface course and the base course, and improving the tensile strength of the pavement. After the appearance of reflective cracks, the transverse crack spacing of the surface layer ranged between 32.8 m and 66.5 m. 15fp-AC25, 15fp-AC20, 15df-AC25, and 17fp-AC25 were found to be the best semi-rigid base asphalt pavement structures to reduce transverse cracking. Finally, transverse cracking in pavement composite structures under different bonding conditions needs to be analyzed in the follow-up work.

## Introduction

Semi-rigid base asphalt pavements have high strength and good stability; thus, they can be applied to heavy traffic. However, transverse cracking is one of the most serious problems for semi-rigid base asphalt pavements. Therefore, it is necessary to investigate how to control or reduce transverse cracking in semi-rigid base asphalt pavements.

In semi-rigid base asphalt pavements, the base course experiences serious cracking due to its temperature shrinkage and dry shrinkage characteristics^1^. The influences of different factors on the shrinkage of the base course of semi-rigid base asphalt pavements have been extensively explored. Cheng et al.^2^ experimentally investigated the dry shrinkage and temperature shrinkage characteristics of a cement-stabilized macadam base with different cement contents and revealed the changes in the dry shrinkage coefficient, the temperature shrinkage stress, and the temperature shrinkage coefficient, providing a basis for the study of shrinkage cracking in semi-rigid base materials. Sharpe^3^ analyzed the dry shrinkage characteristics of cement-stabilized soil and reported that the greater the water loss rate, the higher the drying shrinkage of the base. Feng^4^ experimentally obtained the temperature shrinkage resistances of commonly used semi-rigid base materials, and reported that lime and fly ash–stabilized macadam had the strongest cracking resistance, whereas cement-stabilized macadam had the worst cracking resistance. Wang et al.^5^ analyzed the shrinkage performances of ash macadam with three different gradation types and found that ash macadam with skeleton porosity gradation had the best crack resistance.

The transverse crack spacing is the performance of the degree of transverse cracking of asphalt pavement. Therefore, scholars simulate the transverse cracking of pavement under different service conditions through the calculation model of crack spacing in order to provide reference for pavement design. Rajbongshi et al.^6^ established a temperature stress prediction model for asphalt pavements and the crack spacing was predicted by the stress profile. Prieto-Munoz et al.^7^ analyzed transverse cracking in asphalt pavements based on the theory of plane strain elasticity and established a critical transverse crack spacing model. l, and compare well with finite element results. Yin^8^, based on the energy release rate of asphalt pavement cracks, proposed an open-mode crack initiation, spacing, and saturation calculation method. Timm et al.^9^^,^^10^ established a mechanical model to predict the crack spacings of asphalt concrete pavements and supporting layers under different environmental conditions and material types. Hence, crack spacing calculation models can effectively analyze transverse cracking in asphalt pavements with a semi-rigid base.

After cracking in the base course, the displacement of an asphalt pavement under loading occurs in both horizontal and vertical directions, resulting in the reflection of base cracks to the surface laye^11^. Reflective cracks mainly cause transverse cracking in asphalt pavements. Bao et al.^12^ expounded the influences of the layer thickness and crack spacing of the base course on reflective cracks by numerical simulations and posited that prefabricating cracks with a small spacing in the base course could reduce pavement cracking. Ni et al.^13^ analyzed the propagation of reflective cracks in an asphalt pavement based on the theory of fracture mechanics and reported that the main factors affecting the propagation of reflective cracks were the surface layer material modulus, the surface layer thickness, and the base layer material modulus. Gao^14^ discussed the relationship between crack location and crack type for a semi-rigid base asphalt pavement and noticed that cracks existing 0.2–0.3 m away from the traffic load center in the horizontal direction could more effectively cause reflective cracking of shearing type (mode II cracking in fracture mechanics). Aliha et al.^15^ analyzed the propagation of reflective cracks in an asphalt pavement based on a two-dimensional finite element analysis and found that the thickness and stiffness of the asphalt surface were the main factors affecting the propagation of reflective cracks. It can be seen that materials, structures, loads and other factors cause different degrees of reflection cracks, and reflection cracks are also an important component of transverse cracks. Therefore, reflective cracks cannot be ignored during the analysis of transverse cracking in semi-rigid base asphalt pavements.

Therefore, a calculation model of transverse crack spacing in a semi-rigid base pavement structure was established in the current work. The transverse crack spacing of the asphalt pavement was obtained based on the development process of reflection cracks, and the most suitable structural combination of the semi-rigid base asphalt pavement was recommended. This study could provide a reference for the anti-cracking design of semi-rigid base asphalt pavements.

## Calculation model of transverse crack spacing in a semi-rigid base asphalt pavement

In this section, a semi-rigid base asphalt pavement composite structure is taken as the research object. A calculation model of transverse crack spacing is established based on the shrinkage deformation and stress analyses of the pavement.

### Shrinkage deformation analysis of the pavement structure

**Figure 1:**
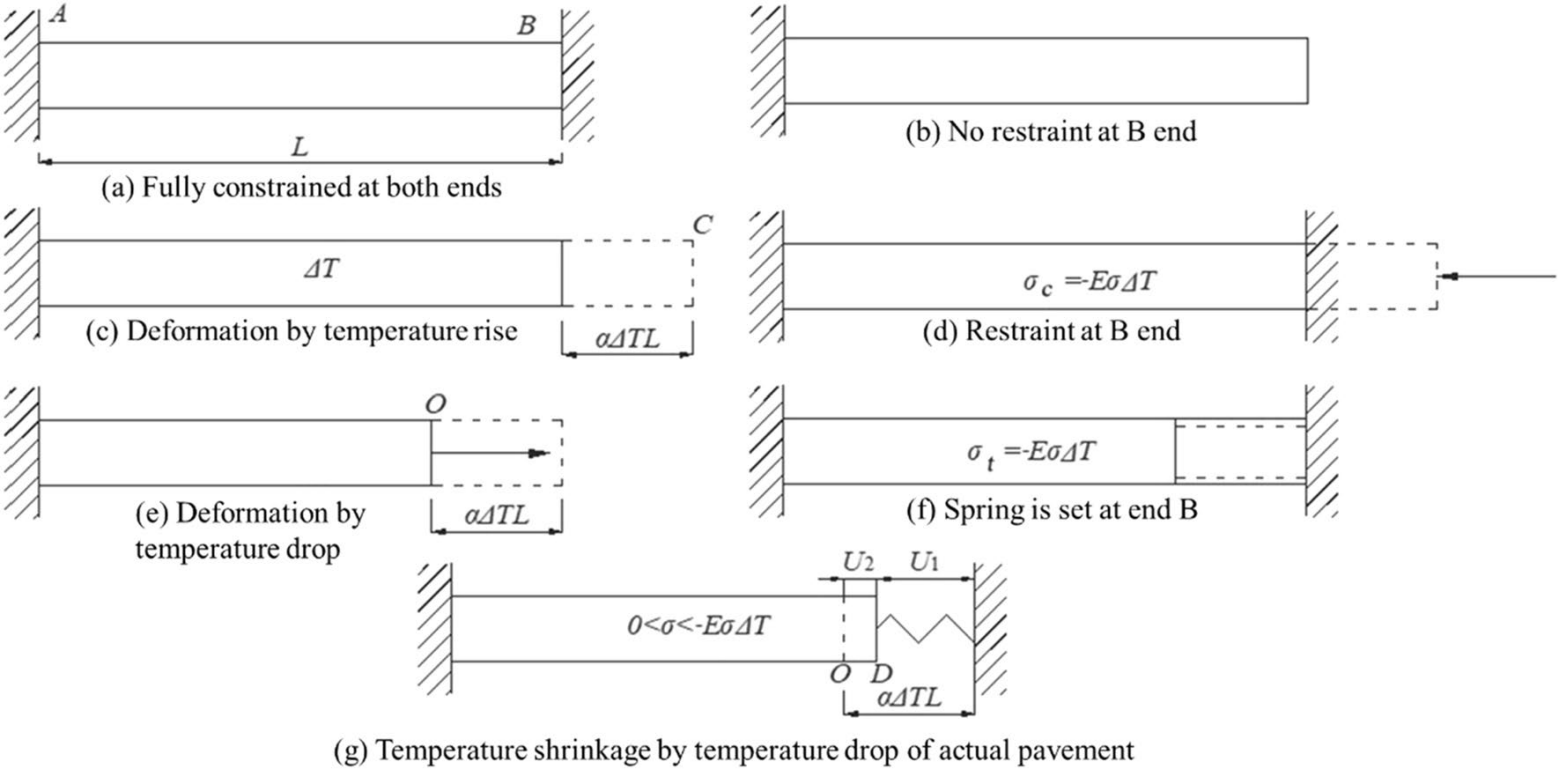
Schematic representation of the temperature shrinkage deformation of the pavement structure.

It is assumed that the surface course and base course of the pavement are closely bonded, and the influence of interlayer slip is not considered. Therefore, each layer of the pavement is regarded as a slab structure (Fig. 1a). When both ends of the thin plate are fully constrained, it could not deform in the length direction. The deformation of the plate in the width direction is ignored. The *B* end of the plate could deform freely after the constrained contact at this end (Fig. 1b). When the plate is subjected to a temperature difference Δ*T*, the free expansion deformation of the plate (*α*Δ*TL*) occurs from *B* to *C* (Fig. 1c), and in this case, the temperature stress in the plate is zero. If there is a complete constraint at the *B* end, the plate could not expand freely, and in this case, the plate uniformly bears the temperature stress (Fig. 1d). According to Hooke's law, the temperature stress can be expressed as

1 $$ \sigma_{c} = - E\alpha \Delta T $$

where *σ*_*c*_ is the compressive stress generated in the plate by temperature, *E* is the elastic modulus, *α* is the shrinkage coefficient, and Δ*T* is the temperature difference (positive).

When there is a temperature difference (Δ*T*) in the plate, temperature shrinkage occurs. If there is no constraint at the *B* end, it shrinks to the *O* end (Fig. 1e), and in this case, the shrinkage deformation of the plate is *α*Δ*TL*. If the plate needs to be restored to its original length, tensile stress should be applied to it (see Fig. 1f), producing uniform temperature stress. According to Hooke's law, the temperature stress can be expressed as

2 $$ \sigma_{t} = - E\alpha \Delta T $$

where *σ*_*t*_ is the tensile stress in the plate caused by temperature and Δ*T* is the temperature difference (negative).

When there is a spring at the *B* end, this end could not freely shrink to the *O* end; however, it becomes stable at the *D* end (Fig. 1g). The actual deformation of the plate consists of free deformation and constrained deformation:

3 $$ U_{1} = U_{2} + \alpha \Delta TL $$

where *U*_*1*_, *U*_*2*_, and *α*Δ*TL* are the actual deformation, constrained deformation, and free deformation of the plate, respectively.

In actual scenarios, when a pavement structure deforms along the length direction, it is constrained by the adjacent area and could not deform freely (Fig. 1g).

**Figure 2:**
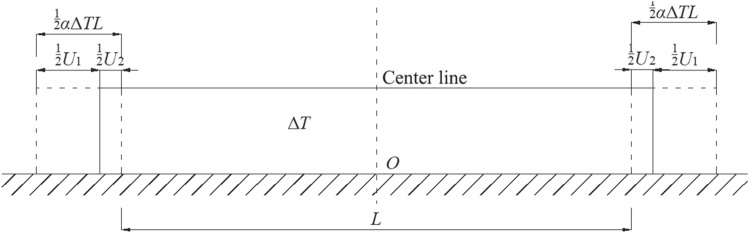
Schematic representation of the continuous elastic constraint.

Moreover, as no strong bonding exists between the base and sub-base of a pavement, the interlayer slip between them cannot be ignored. Furthermore, the constraint between the base and the sub-base can be regarded as a continuous elastic constraint. Under the action of this continuous elastic constraint, the pavement structure experiences both free deformation and constrained deformation (Fig. 2).

In the above analysis, the internal temperature of the pavement structure is kept constant. However, in actual scenarios, the temperature change in a pavement structure is not coordinated. For example, when the ambient temperature drops rapidly, the temperature of the upper part of the pavement structure also changes rapidly. The temperature inside the pavement structure is less affected by ambient temperature. Thus, the temperature changes slowly, causing a temperature difference inside the pavement. In addition, the temperature shrinkage coefficients of the base course and the surface layer are different because of their different material properties. Therefore, it is necessary to calculate the temperature difference inside the pavement structure and the temperature shrinkage coefficients of different structural layers.

### Calculation model of transverse crack spacing

According to the research on pavement structural strain by Chen^16^ and Wu et al.^17^, the strain of the pavement composite structure along the height can be calculated as

4 $$ \varepsilon_{\alpha t} \left( y \right) = \varepsilon_{0} + \psi y $$

where *y* is the height from the bottom of the sub-base of the pavement structure, *ε*_0_ is the strain at *y* = 0, and *ψ* is the flexural curvature of the pavement composite structure after temperature shrinkage deformation.

The deformation along the height of the pavement composite structure can be calculated as

5 $$ \Delta l\left( y \right) = l \, \varepsilon_{\alpha t} \left( y \right) = l(\varepsilon_{0} + \psi y) $$

where *l* is the pavement length.

The temperature shrinkage coefficient along the height of the pavement structure can be obtained as

6 $$ \alpha_{t} \left( y \right) = \varepsilon_{\alpha t} \left( y \right)/T(y) $$

where *α*_*t*_(*y*) is the temperature shrinkage coefficient of each layer.

According to research findings of Shi et al.^18^ and Zhang et al.^19^, the maximum shrinkage stress of the surface layer of the pavement structure can be determined by

7 $$ \sigma_{\max } = E\left( y \right)\alpha \left( y \right)\Delta T\left( {\frac{1}{{{\text{cosh}}K\frac{L}{2}}} - 1} \right) $$

where *σ*_*max*_ is the maximum shrinkage stress of the surface layer, *E*(*y*) is the elastic modulus of the pavement structure (the elastic moduli of the base layer and the surface layer are *E*_b_ and *E*_sf_, respectively), *L* is the crack spacing, *T* is the cooling range; *α*_*t*_(*y*) is the temperature shrinkage coefficient of each structural layer (the temperature shrinkage coefficients of the base layer and the surface layer are *α*_bt_ and *α*_sft_, respectively). For concise, *K* is introduced and calculated by Eq. (8):

8 $$ K = \sqrt {\frac{{C_{x} }}{E\left( y \right)h\left( y \right)}} $$

where *C*_*x*_ is the friction resistance coefficient between the sub-base and the base of the pavement structure, and its value generally ranges between 0.6 ~ 1.2 N/mm^3^, *h*(*y*) is the thickness of each structural layer (the thicknesses of the base layer and the surface layer are *h*_b_ and *h*_sf_, respectively).

The maximum dry shrinkage stress can be calculated as

9 $$ \sigma_{d\max } = E_{{\text{b}}} \alpha_{{{\text{b}}d}} \omega \left( {\frac{1}{{{\text{cosh}}K_{{{\text{b}}d}} \frac{L}{2}}} - 1} \right) $$

where *α*_*bd*_ and *ω* are the drying shrinkage coefficient and water loss rate of the base course, respectively.

As shown in Eq. (9), the maximum tensile stress is proportional to the length and elastic modulus. Assuming that the elastic modulus is certain, if the length range is certain, the maximum tensile stress can be guaranteed not to exceed the ultimate tensile stress.

According to the concrete strength theory, it is assumed that the stress–strain relationship of cement stabilized macadam is linear before failure, namely:

10 $$ \sigma_{d\max } { = }\varepsilon_{p} E_{b} $$

11 $$ \varepsilon_{p} E_{b} = E_{{\text{b}}} \alpha_{{{\text{b}}d}} \omega \left( {\frac{1}{{{\text{cosh}}K_{{{\text{b}}d}} \frac{L}{2}}} - 1} \right) $$

12 $$ L_{{{\text{sf}}\max 1}} = \frac{2}{{K_{{{\text{sf}}}} }}{\text{acosh}}\frac{{\alpha_{{{\text{sf}}}} \Delta T}}{{\varepsilon_{{{\text{sf}}}} { + }\alpha_{{{\text{sf}}}} \Delta T}} $$

*L*_sfmax1_ is inversely calculated according to Equation. That is, the maximum spacing without cracks is obtained.

When temperature drops, △*T* is negative, *ε*_*sf*_ is positive and vice versa. Therefore, *α*_*sf*_ is opposite to *ε*_*sf*_. Therefore, the crack spacing of the surface layer can be determined as

13 $$ L_{{{\text{sf}}\max 1}} = \frac{2}{{K_{{{\text{sf}}}} }}{\text{acosh}}\frac{{\left| {\alpha_{{{\text{sf}}}} \Delta T} \right|}}{{\left| {\alpha_{{{\text{sf}}}} \Delta T} \right| - \left| {\left[ {\varepsilon_{{{\text{sf}}}} } \right]} \right|}} $$

where *L*_sf max 1_ is the spacing of surface cracks, and [*ε*_sf_] is the ultimate tensile strain of the surface layer.

When the surface layer cracks, the maximum crack spacing of the pavement is reduced by half. Thus, the crack length of the pavement can be expressed as

14 $$ L_{{{\text{sf}}\max 2}} = \frac{1}{2}L_{{{\text{sf}}\max 1}} $$

The average crack spacing of the surface layer can be determined as

15 $$ \overline{L}_{{{\text{sf}}}} = \frac{1}{2}\left( {L_{{{\text{sf}}\max 1}} + L_{{{\text{sf}}\max 2}} } \right) = \frac{3}{4}L_{{{\text{sf}}\max 1}} $$

The crack spacing at the base layer can be obtained as

16 $$ L{ = }\frac{2}{{K_{{\text{b}}} }}{\text{acosh}}\frac{{\alpha_{{{\text{bt}}}} \Delta T + \alpha_{{{\text{b}}d}} \omega }}{{\left[ {\varepsilon_{{\text{b}}} } \right] + \alpha_{{{\text{bt}}}} \Delta T + \alpha_{{{\text{b}}d}} \omega }} $$

where *α*_bd_ and [*ε*_b_] are the drying shrinkage coefficient and ultimate tensile strain of the base, respectively.

The premise conditions of Eq. (16) are that the shrinkage stress of the base course is equal to its ultimate tensile stress and there is no cracking in the base layer. When the shrinkage stress of the base layer exceeds the ultimate tensile stress, it cracks and the crack spacing is reduced by half; thus,

17 $$ L_{{{\text{b}}\max 2}} = \frac{1}{2}L_{{{\text{b}}\max 1}} $$

The average crack spacing of the base can be calculated as

18 $$ \overline{L}_{{\text{b}}} = \frac{1}{2}\left( {L_{{{\text{b}}\max 1}} + L_{{{\text{b}}\max 2}} } \right) = \frac{3}{4}L_{{{\text{b}}\max 1}} $$

When the material parameters of each layer of the pavement structure are known, the crack spacing of each layer could be estimated according to the above equations.

## Parameter acquisition for the transverse crack spacing calculation model

In order to predict cracking in the semi-rigid base asphalt pavement, it is necessary to obtain the elastic moduli and shrinkage coefficients of the surface layer and base layer materials according to the aforesaid calculation model.

### Specimen preparation

**Figure 3:**
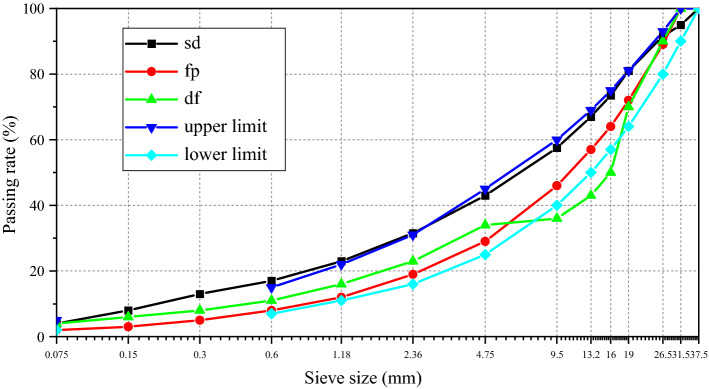
Gradation curves of lime and fly ash-stabilized macadam.

The semi-rigid base material contained lime and fly ash-stabilized macadam, andesite aggregates (produced in Heilongjiang Province), lime (produced by the Jilin Chemical Industry Company), and Class I fly ash (produced by the Harbin Power Plant), and it met all Technical Specifications for Construction of Highway Roadbases (JTJ 034-2000 standard). The mass ratio of lime to fly ash was 1:3. Lime and fly ash–stabilized macadam had three types of gradation: suspension dense gradation (sd), framework pore gradation (fp), and dense framework gradation (df), and the corresponding gradation curves are displayed in Fig. 3. The total contents of lime and fly ash in lime and fly ash-stabilized macadam were 15%, 17%, and 20%, respectively. The maximum dry density and optimum water content of lime and fly ash-stabilized macadam under different gradations are listed in Table 1. The sample size was 100 mm × 100 mm × 400 mm. The samples were molded by vibration compaction and then cured according to the standard curing method. Three test specimens should be prepared for each mixture for parallel test.

**Table 1:** Maximum dry density and optimum moisture content of lime and fly ash-stabilized macadam.

Material type	15 sd	15 fp	15 df	17 sd	17 fp	17 df	20 sd	20 fp	20 df
Maximum dry density (g/cm^3^)	2.23	2.32	2.38	2.24	2.33	2.27	2.27	2.28	2.30
Optimum moisture content (%)	6.06	5.77	5.87	6.99	5.55	6.04	7.13	6.00	6.36

**Figure 4:**
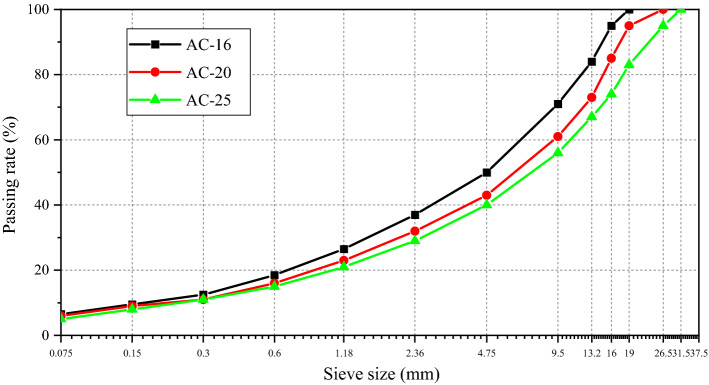
Gradation curves of different asphalt concrete mixtures.

In the pavement structure, the subsurface layer was directly bonded with the base layer; thus, AC-16, AC-20, and AC-25 were used as surface materials. The concrete contained 90# base asphalt of PetroChina, andesite aggregates (produced in Heilongjiang Province), and limestone mineral powder (produced in Jilin Province), and it meets all Technical Specifications for Construction of Highway Asphalt Pavements (JTG F40-2017 standard). The grading curves of the three asphalt mixtures are displayed in Fig. 4, and the optimum asphalt contents for AC-16, AC-20, and AC-25 were 4.5%, 4.3%, and 3.9%, respectively. The specimen size was 60 mm × 100 mm × 400 mm, and.

### Shrinkage coefficient of the base course material

#### Dry shrinkage coefficient

**Figure 5:**
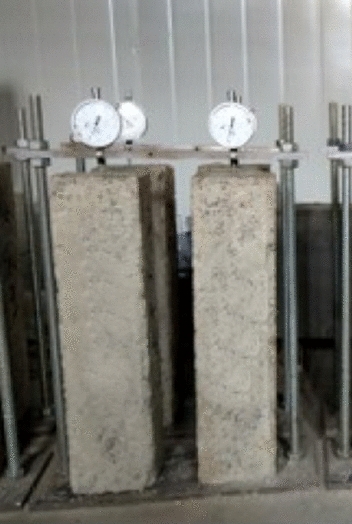
Dry shrinkage coefficient test setup of lime and fly ash-stabilized macadam.

**Figure 6:**
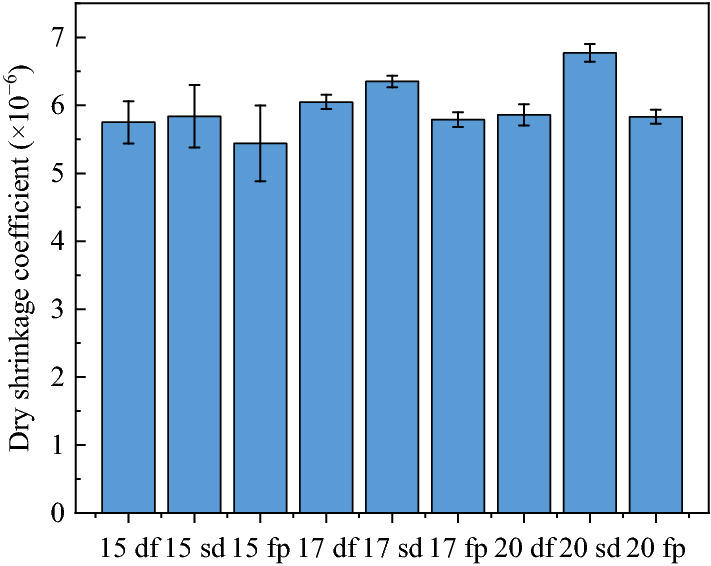
Dry shrinkage coefficients of lime and fly ash-stabilized macadam.

The drying shrinkage coefficient of lime and fly ash–stabilized macadam was measured according to the Test Method of Materials Stabilized with Inorganic Binders for Highway Engineering (JTG E51-2009 standard). The sample with size of 100 mm × 100 mm × 400 mm was molded by vibration compaction, and then cured according to the standard curing method. After the surface of the saturated specimen was wiped, the initial length of the specimen should be measured. A glass sheet was stuck on the specimen long end section with *α*-cyanoacrylate. The dial indicator was fixed on the shrinkage indicator (Fig. 5). The specimen was placed in the drying shrinkage chamber. The reading of the dial indicator was recorded every day. The test lasted for 31 days. The drying shrinkage coefficients of the lime and fly ash–stabilized macadam base are presented in Fig. 6.

the samples were compacted by vibration. Three parallel specimens were prepared for each mixture.

#### Temperature shrinkage coefficient

The temperature shrinkage coefficient of the base course material was determined by gradually decreasing the temperature. However, this process was relatively complicated, and the result was affected by the shrinkage of the metal stent. Zhao^20^ measured the temperature shrinkage coefficients of lime and fly ash-stabilized macadam, asphalt concrete, and other pavement materials based on the temperature rising method and ignored the error caused by the shrinkage of metal supports. Yang et al.^21^ and Tian et al.^22^ asserted that the temperature shrinkage characteristics of materials during heating and cooling were similar and the temperature shrinkage and expansion coefficients were very close. Therefore, in this work, the temperature rise method was used to measure the temperature shrinkage coefficient of lime and fly ash-stabilized macadam.

**Figure 7:**
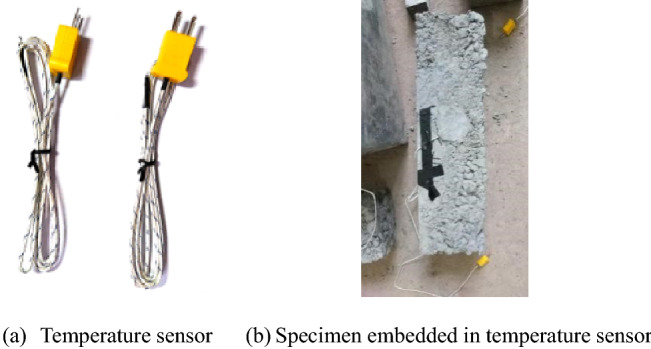
Temperature sensor and specimen to be tested.

A temperature sensor was embedded inside each specimen (Fig. 7). The temperature range for the shrinkage test was − 30 °C ~ 18 °C. The specimen was first dried and cooled to room temperature. A glass sheet was stuck on the specimen surface with *α*-cyanoacrylate. The specimen was then kept in a freezer at − 30 °C for three hours. After removing from the freezer, the specimen was placed on a metal bracket equipped with a dial indicator. The temperature of the specimen and the reading of the dial indicator were collected every five minutes, and when values became the same, the test was terminated.

**Figure 8:**
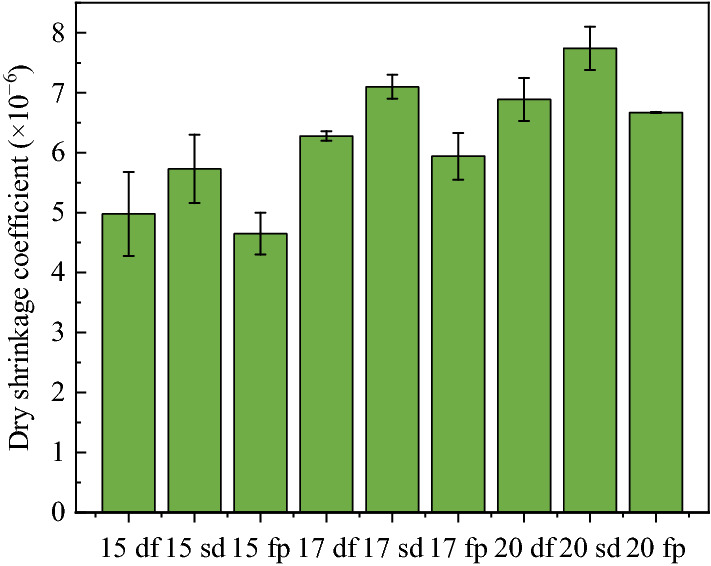
Temperature shrinkage coefficients of the lime and fly ash-stabilized macadam base.

The temperature shrinkage coefficients of lime and fly ash-stabilized macadam are presented in Fig. 8.

### Shrinkage coefficient of the surface course material

**Figure 9:**
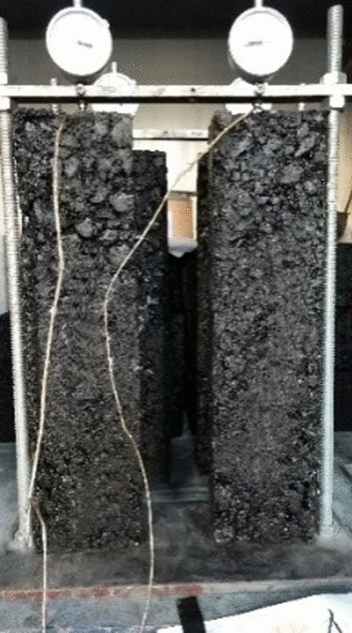
Temperature shrinkage coefficient test setup for asphalt concrete.

**Figure 10:**
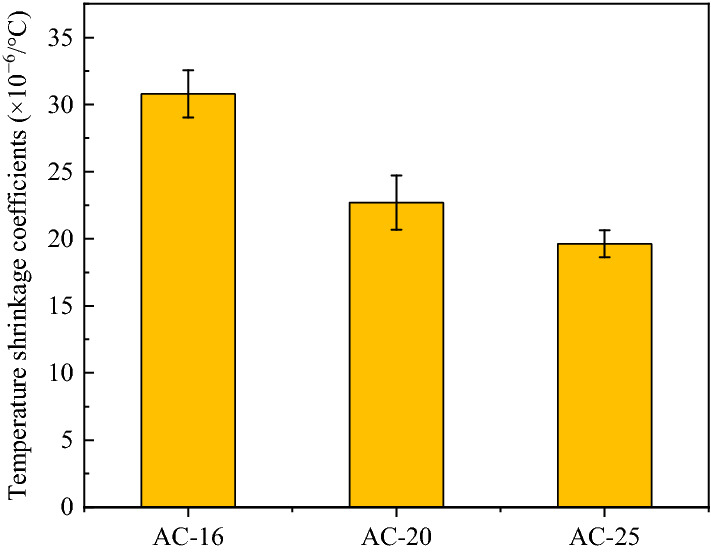
Temperature shrinkage coefficients of asphalt concrete.

The temperature shrinkage coefficient of the asphalt concrete pavement was measured by the temperature rise method. The temperature range was − 30 °C ~ 18 °C, and the test setup is exhibited in Fig. 9. The temperature shrinkage coefficients of asphalt concrete are presented in Fig. 10.

As the asphalt concrete surface layer and the lime and fly ash-stabilized macadam base formed a composite structure, they influenced each other in the shrinkage process. Therefore, the shrinkage coefficient of the pavement composite structure needs to be measured.

In order to ensure effective bonding between the surface layer and the base layer, Styrene Butadiene Styrene (SBS) modified asphalt was used as the binder and the coating thickness was set to 1 mm^23^. After bonding, a heavy object was placed on top of the surface layer of the pavement composite structure, and the surface layer and the base layer should be static pressed with 50 kg steel plate for 24 h to bond firmly.

**Figure 11:**
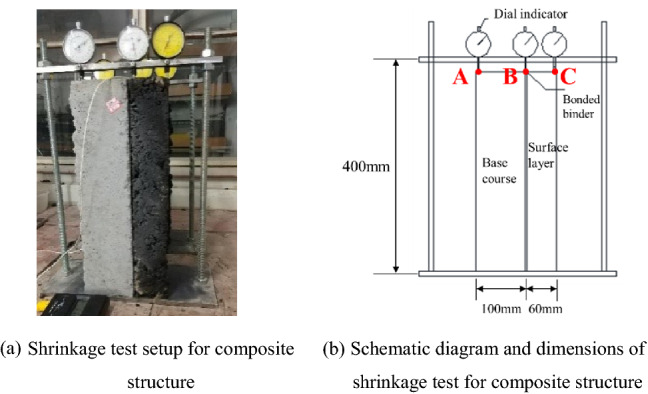
Shrinkage coefficient measurement setup for the pavement composite structure.

The shrinkage coefficient of the pavement composite structure was measured by the temperature rise method. The temperature range was −30 °C–8 °C. The size of composite structure for testing is 160 mm × 100 mm × 400 mm. A dial indicator was set at the bottom of the base (A), at the bonding site (B), and also at the top of the surface layer (C) (Fig. 11), and the test results are presented in Table 2.

**Table 2:** Shrinkage coefficients of the semi-rigid base asphalt pavement composite structure.

Structural combination type	Temperature shrinkage coefficient (× 10^−6^/°C)		
	Bottom of the base course (A)	Bonding site (B)	Top of the surface course (C)
15 df-AC16	7.79	16.22	20.52
15 df-AC20	7.21	14.19	18.39
15 df-AC25	6.95	12.71	16.93
15 sd-AC16	9.19	17.68	21.74
15 sd-AC20	8.62	14.77	19.17
15 sd-AC25	7.89	13.05	17.71
15 fp-AC16	7.14	16.61	18.54
15 fp-AC20	6.17	13.96	16.59
15 fp-AC25	5.60	12.76	15.34
17 df-AC16	9.22	18.28	20.94
17 df-AC20	8.75	14.95	19.11
17 df-AC25	7.37	13.36	17.60
17 sd-AC16	10.05	17.84	20.76
17 sd-AC20	9.19	15.55	19.61
17 sd-AC25	8.26	13.28	17.11
17 fp-AC16	8.67	16.77	19.71
17 fp-AC20	8.05	14.51	18.36
17 fp-AC25	7.40	13.41	16.98
20 df-AC16	10.08	18.52	21.35
20 df-AC20	9.53	15.47	20.03
20 df-AC25	8.23	13.88	18.36
20 sd-AC16	10.68	19.30	22.16
20 sd-AC20	10.00	16.46	20.42
20 sd-AC25	9.06	15.03	18.57
20 fp-AC16	9.66	18.10	20.96
20 fp-AC20	8.39	15.76	19.61
20 fp-AC25	7.58	14.61	18.02

## Results and discussion

### Transverse crack spacing in the pavement composite structure

The transverse crack spacings of the semi-rigid base asphalt pavement under different structural combinations were compared. From the calculation model of transverse crack spacing, it can be seen that the transverse crack spacing of composite structure is related to the shrinkage coefficient, resilience moduli, ultimate tensile strains, resulting layer thickness, friction coefficient between the base and the sub-base and cooling ranges. According to *Technical Rules for Frost Resistance Design and Construction of Highway Engineering*, the initial temperature was set to 18 °C, and the lowest temperatures were − 20 °C, − 25 °C, − 30 °C, − 35 °C, − 37 °C; thus, the corresponding cooling ranges were 38 °C, 43 °C, 48 °C, 53 °C, and 55 °C, respectively. The thicknesses of the surface layer (*h*_sf_) and the base layer (*h*_b_) were 0.15 m and 0.35 m, respectively. The ultimate tensile strains of the surface layer ([*ε*_sf_]) and the base layer ([*ε*_b_]) at low temperatures were 5.0 × 10^−4^ and 2.0 × 10^−4^, respectively^24^. The friction coefficient (*C*_*x*_) between the base and the sub-base was 0.9 N/mm^3^. The resilience moduli of the surface layer and base layer of the pavement structure were measured according to the Test Methods of Materials Stabilized with Inorganic Binders for Highway Engineering (JTG E51-2009, JTG E20-2011). For the asphalt surface layer, the resilience modulus was measured by cylinder method. The asphalt concrete specimen was cylinder with the dimension of 100 mm and height of 100 mm. After heat preservation in water bath for 2.5 h. The readings of dial indicator and actual load were recorded for each loading and unloading process. For the semi-rigid base, the resilience modulus was measured by topside method. The specimens were cured according to the standard method. The end face should be plastered with cement slurry and sprinkled with a small amount of fine sand. The dial indicator was set on the top surface after the specimen was immersed in water for 24 h. The reading of the dial indicator should be recorded after each loading and uploading. The corresponding results are presented in Table 3.

**Table 3:** Resilience moduli of the surface layer and the base layer (MPa).

Structural layer	Material type	Cooling range (°C)				
		38	43	48	53	55
Base course	15 sd	1347	1374	1402	1430	1441
	15 df	1766	1822	1878	1934	1956
	15 fp	1478	1509	1540	1572	1584
	17 sd	1612	1648	1683	1719	1733
	17 df	2045	2090	2135	2181	2199
	17 fp	1541	1591	1642	1692	1712
	20 sd	1878	1928	1978	2028	2048
	20 df	2223	2269	2316	2362	2381
	20 fp	1565	1600	1636	1671	1685
Surface course	AC-16	8342	10239	12569	15429	16747
	AC-20	7998	9818	12052	14794	16059
	AC-25	7669	9414	11557	14186	15398

#### Transverse crack spacing in the base course

**Figure 12:**
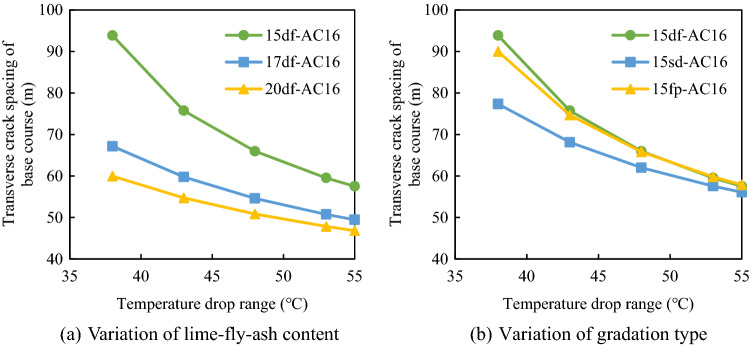
Variation of transverse crack spacing in the base course with the cooling range.

The variation of transverse crack spacing in the base course with the cooling range was determined according to the above parameters, and the corresponding results are displayed in Fig. 12.

It is noticeable from Fig. 12 that for the lime and fly ash-stabilized macadam base, when the cooling range increased from 38 to 55 °C, the transverse crack spacing gradually decreased. For example, when the temperature dropped by 38 °C, the crack spacing of the 15df-AC-16 composite structure was 93.86 m. However, when the temperature dropped by 55 °C, the crack spacing was only 57.52 m, the number of cracks nearly doubled. Figure 12a reveals that the crack spacing of the pavement structure decreased with the increase in the content of lime and fly ash. For example, when the temperature drop was 38 °C, the crack spacings of the composite structure were only 67.47 m and 59.97 m for the lime and fly ash contents of 17% and 20%, respectively, reflecting that the lime and fly ash content greatly affected the spacing of transverse cracks. The reason lies in that the higher content of lime-fly-ash, the greater the drying shrinkage deformation and temperature shrinkage deformation. Therefore, for the lime and fly ash–stabilized macadam base, the dosage of lime and fly ash should be appropriately reduced to control the crack spacing.

Figure 12b indicates that the gradation type of the base also affected the transverse crack spacing in the base. The df-grade base had the largest transverse crack spacing, followed by the fp-grade base and the sd-grade base. This is because the principle of skeleton dense structure interlocking can ensure the aggregate more dense, and the internal friction and the cohesion of the base material are higher. Therefore, the suspended dense gradation should be avoided for the base layer of the pavement structure.

#### Transverse crack spacing in the surface course

**Figure 13:**
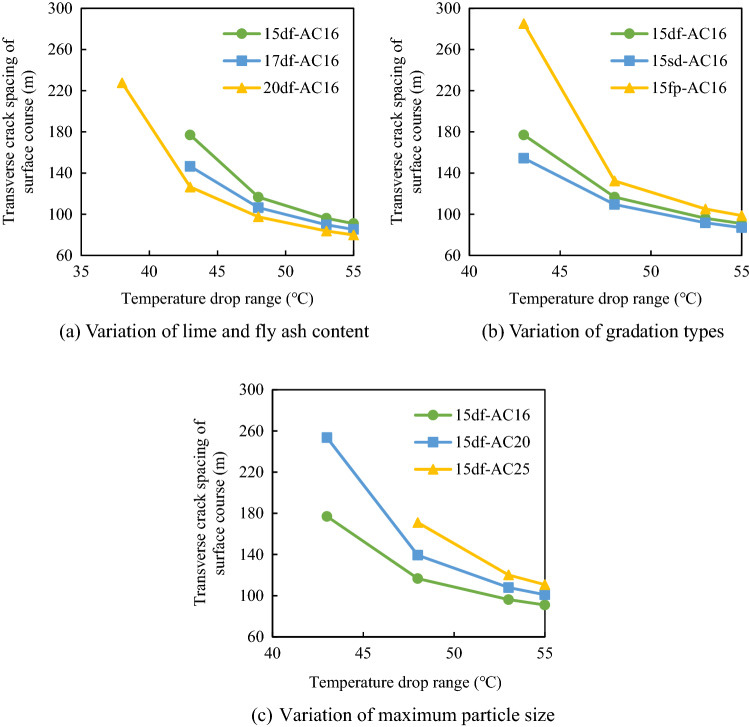
Variation of the transverse crack spacing in the surface layer with the cooling range.

The type of the base course and the surface course affected the transverse crack spacing in the surface course. Figure 13 exhibits the relationship between the transverse crack spacings of different composite structures and the cooling range.

It is evident from Fig. 13 that the crack spacing in the surface layer of the pavement composite structure decreased with the increase in the cooling range. When the temperature was higher than − 30 °C (cooling range was less than 48 °C), the transverse crack spacing of the surface layer first decreased sharply and then dropped slowly with the increase in the cooling range. For example, when the temperature-lowering amplitude decreased from 43 to 48 °C, the crack spacing of the 15df-AC-16 composite structure decreased from 177 to 116.7 m. However, when the cooling range increased from 48 to 55 °C, the crack spacing was only reduced by 26 m. When the spacing of transverse cracks was reduced to a certain extent, the temperature stress in the asphalt pavement was released.

Moreover, when the lime and fly ash content of the macadam base changed, the spacing of transverse cracks in the pavement also changes. The lower the content of lime and fly ash, the greater the spacing of transverse cracks on the surface layer (Fig. 13a). For example, when the temperature drop was 43 °C, the fracture spacings for the lime and fly ash dosages of 15%, 17%, 20% were 177 m, 146.5 m, and 126.5 m, respectively. The smaller the content of lime and fly ash, the smaller the temperature shrinkage coefficient of the base course and the greater the inhibition effect on the surface course shrinkage; thus, increasing the transverse crack spacing and reducing the number of cracks. Therefore, to control the transverse crack spacing in the surface layer, the dosage of lime and fly ash in the macadam base should be appropriately reduced.

Moreover, the transverse crack spacing in the surface layer was also affected by the gradation type of the base layer. When the base gradation was the framework pore gradation, the transverse crack spacing of the composite structure was larger than those for the other two base gradations and the number of transverse cracks was the lowest (Fig. 13b). Therefore, in order to control the crack spacing of the surface layer, the skeleton gap gradation could be used for the base.

The gradation type of the pavement also had a great influence on transverse crack spacing (Fig. 13c. The larger the nominal maximum aggregate size in the subsurface layer, the larger the transverse crack spacing. For example, when the temperature drop range was 48 °C, the crack spacings of the AC-25 and AC-16 surface layers were 171 m and 116.7 m, respectively. Therefore, asphalt concrete with a larger nominal maximum aggregate size could be used in the surface layer of the pavement structure.

In addition, the crack spacing of the surface layer was found to be larger than that of the base layer for the same composite structure. For example, when the temperature dropped by 55 °C, the transverse crack spacing in the surface layer of the 20sd-AC-16 composite structure was 80 m, whereas that in the base layer was only 47 m. It happened because the shrinkage and cracking in the base layer occurred under the synergistic effect of temperature shrinkage and dry shrinkage. Moreover, the ultimate tensile strain of the base layer at low temperatures was smaller than that of the surface layer; thus, the spacing of transverse cracks was smaller, and the number of cracks was higher.

### Factors affecting transverse crack spacing

The transverse crack spacing in the pavement structure was affected by numerous factors: the shrinkage coefficients of the base and surface layer materials, the friction resistance coefficient between the base and the sub-base, the tensile strength of the base, and the thickness of pavement structural layers.

#### Effect of shrinkage coefficient on transverse crack spacing

**Figure 14:**
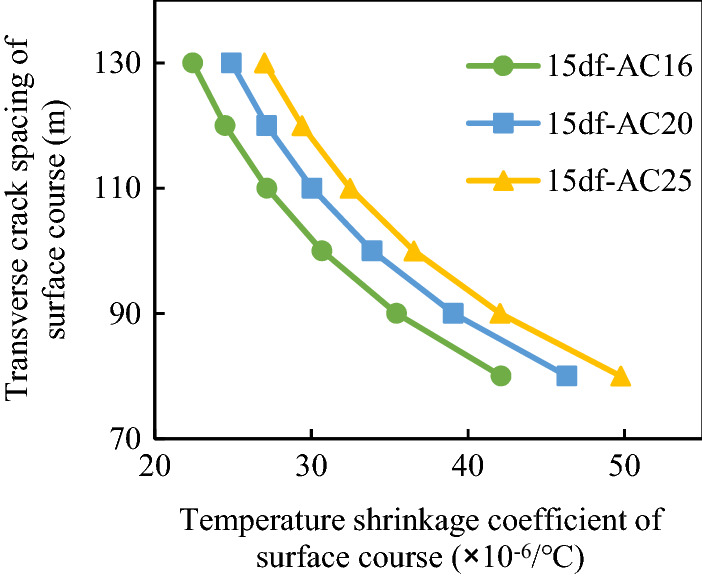
Relationship between transverse crack spacing and surface temperature shrinkage coefficient.

**Figure 15:**
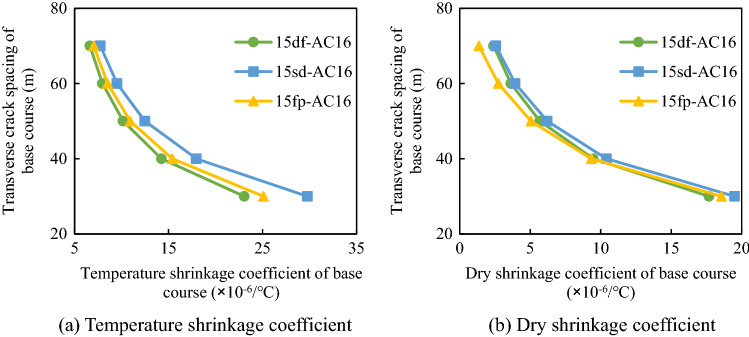
Relationship between the transverse crack spacing and shrinkage coefficients of the semi-rigid base.

The transverse crack spacing of the composite pavement structure was affected by the temperature shrinkage coefficient, whereas that of the semi-rigid base was affected by both the temperature shrinkage coefficient and the dry shrinkage coefficient. The influence of the shrinkage coefficient on the transverse crack spacing in different composite structures was analyzed. The temperature drop range was 48 °C. The thickness and ultimate tensile strain of the surface layer were 0.15 m and 5.0 × 10^−4^, respectively, and the corresponding values for the base course were 0.35 m and 2.0 × 10^−4^, respectively. The friction coefficient between the base and the sub-base was 0.9 N/mm^3^. The elastic moduli of the surface and base layer materials were determined according to Table 2. The variations of the transverse crack spacing of different composite structures with the shrinkage coefficient are displayed in Figs. 14 and 15.

Figure 14 shows that with the increase in the temperature shrinkage coefficient of the surface course, the spacing of transverse cracks gradually decreased. Figure 15 indicates that for the semi-rigid base, with the increase in the dry shrinkage coefficient and the temperature shrinkage coefficient, the transverse crack spacing gradually decreased. The reason is that the larger shrinkage coefficient of the material will bring larger shrinkage deformation, thus reducing the crack spacing. Therefore, during the design of semi-rigid base asphalt pavement structures, attention should be paid to reducing the temperature shrinkage coefficient of the surface layer and controlling the drying shrinkage coefficient and temperature shrinkage coefficient of the semi-rigid base.

#### Effect of pavement structural layer thickness on transverse crack spacing

**Figure 16:**
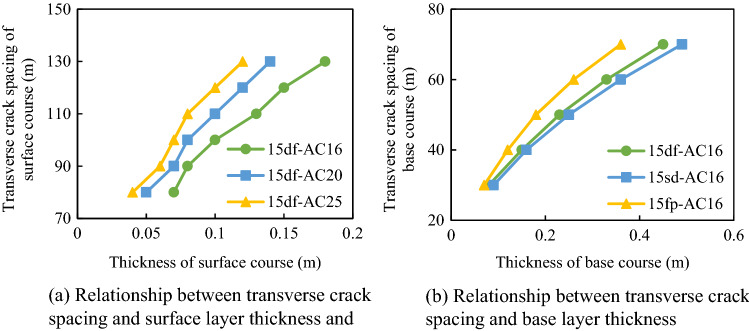
Relationship between transverse crack spacing and structural layer thickness of the pavement composite structure.

To analyze the influence of structural layer thickness on transverse crack spacing, crack spacings for different structural layer thicknesses were calculated (Fig. 16).

The spacing of transverse cracks increased gradually with the increase in the base layer and surface layer thicknesses. The resistance to transverse cracking got strong with the decrease of the thickness of the structural layer. Therefore, the lateral cracking resistance of the pavement could be improved by increasing the thickness of structural layers. Meanwhile, with the increase in the structural layer thickness, the project cost will also simultaneously increase. Therefore, it is necessary to find a balance between the structural layer thickness and the project cost to achieve a better anti-lateral cracking effect.

#### Effect of the tensile strength of the pavement on transverse crack spacing

**Figure 17:**
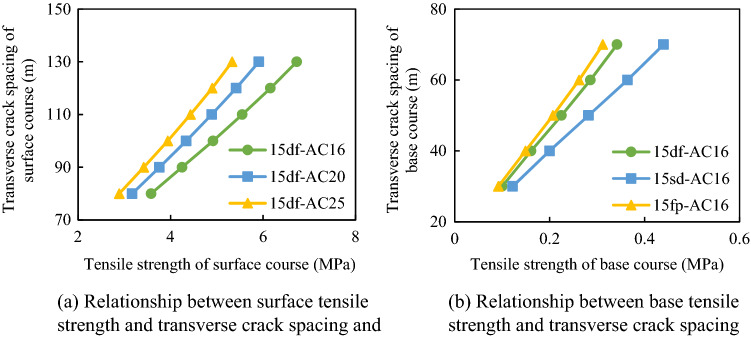
Relationship between the tensile strength of the pavement composite structure and transverse crack spacing.

Transverse cracking was closely related to the tensile strength of the pavement structure. The relationships between the transverse crack spacing and tensile strength of different composite structures are presented in Fig. 17.

It can be seen in Fig. 14 that the crack spacings of the base layer and the surface layer gradually increased with the increase in the tensile strength of the pavement. Moreover, for the asphalt concrete, when the tensile strength of the surface layer was greater than 5.5 MPa, the transverse crack spacing could reach 130 mm. Generally, the tensile strength of asphalt concrete could reach 8.8 MPa at − 30 °C^25^. Therefore, at − 30 °C, the use of asphalt concrete as the surface layer material could keep the transverse crack spacing in the surface course at a large level.

### Calculation of transverse crack spacing considering reflective cracks

When cracks appear in the base, under the action of vehicle load, the surface layer also cracks, causing the appearance of reflective cracks. Therefore, the influence of reflective cracks on transverse cracking was discussed.

#### Appearance of reflective cracks

**Figure 18:**
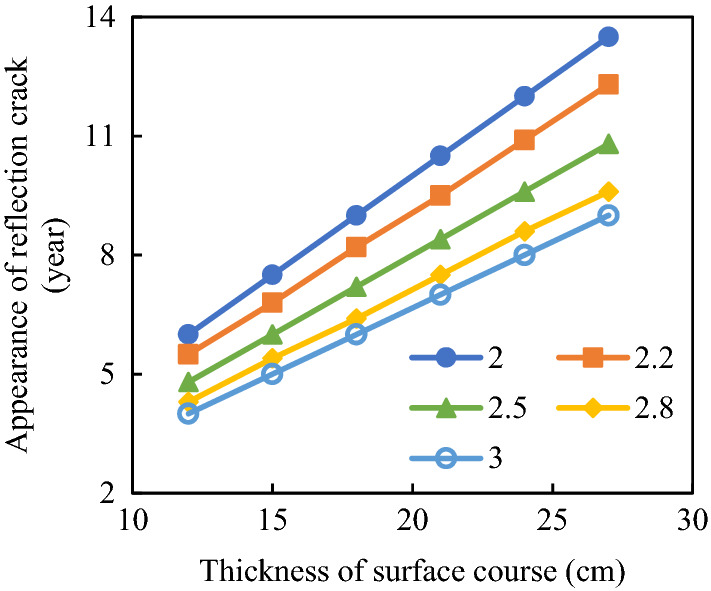
Reflective crack appearance times for different pavement thicknesses.

Growth rates of reflective cracks in asphalt pavements fluctuate around 2.5 cm/year^26^. Considering the difference in reflective crack propagation rates in different environments, the reflective crack appearance times for different pavement thicknesses were calculated (Fig. 18).

With the increase in the crack propagation speed, the appearance time for reflective cracks on the surface layer was gradually shortened. For instance, for the pavement thickness of 15 cm, when the crack propagation speed is 2 cm/year, reflective cracks would appear in the surface layer in 7.5 years. However, when the crack propagation speed of the base layer is 3 cm/year, the appearance time of reflective cracks on the surface layer would appear in the fifth year. However, with the increase in the surface layer thickness, the appearance time of reflective cracks would be prolonged. When the crack propagation speed is 3 cm/year and the surface layer thickness is 24 cm, reflective cracks would appear in the eighth year. Therefore, the crack propagation rate and the surface layer thickness greatly affect the appearance time of reflective cracks in asphalt pavements. To delay the appearance of reflective cracks in asphalt pavements, both the thickness and anti-cracking performance of asphalt pavements need to be improved.

To explore the appearance time of reflective cracks in different pavement structures, AC-16 and AC-20 asphalt mixtures were taken as experimental objects and the appearance time of reflective cracks was analyzed based on their fatigue properties. The fatigue temperature was 15 °C, and the loading frequency was 10 Hz. We revised the fatigue test results with reference to the research in^27,28^. The intermittent load time coefficient was 5, the crack propagation influence coefficient was 20, the lateral distribution coefficient of the driving load was 1/0.5, the adverse season influence coefficient was 365/60, and the total influence coefficient was 1216.67, and the corresponding fatigue test results are presented in Table 4. The fatigue life of AC-16 was found to be longer than that of AC-20, and the crack propagation seed was relatively slow.

**Table 4:** Fatigue test results of different asphalt mixtures.

Material type	Fatigue life	Corrected fatigue life	Crack propagation rates (× 10^−6^ mm/cycle)
AC-16	17589	21399429	1.40
AC-20	15714	19119048	1.57

**Figure 19:**
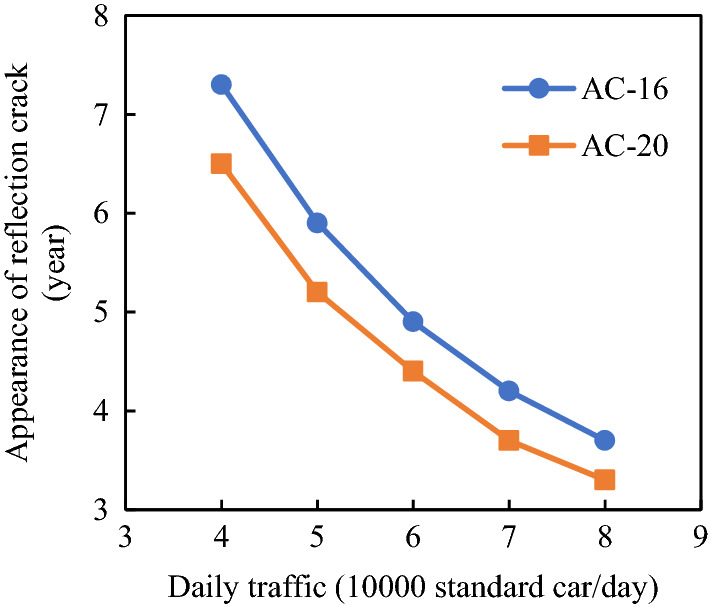
Traffic volume and reflective crack appearance time (Surface layer thickness = 15 cm).

According to the crack propagation speeds of AC-16 and AC-20, the times required for base cracks to reflect to the surface layer under different traffic conditions were calculated (Fig. 19).

With the increase in daily traffic, the appearance time of reflective cracks was gradually reduced, indicating that for the pavement with a general traffic volume, reflective cracks would appear after several years of traffic. If the daily traffic volume is 60,000 standard passenger cars per day, reflective cracks would appear within five years.

#### Calculation of transverse crack spacing considering reflective cracks

**Figure 20:**
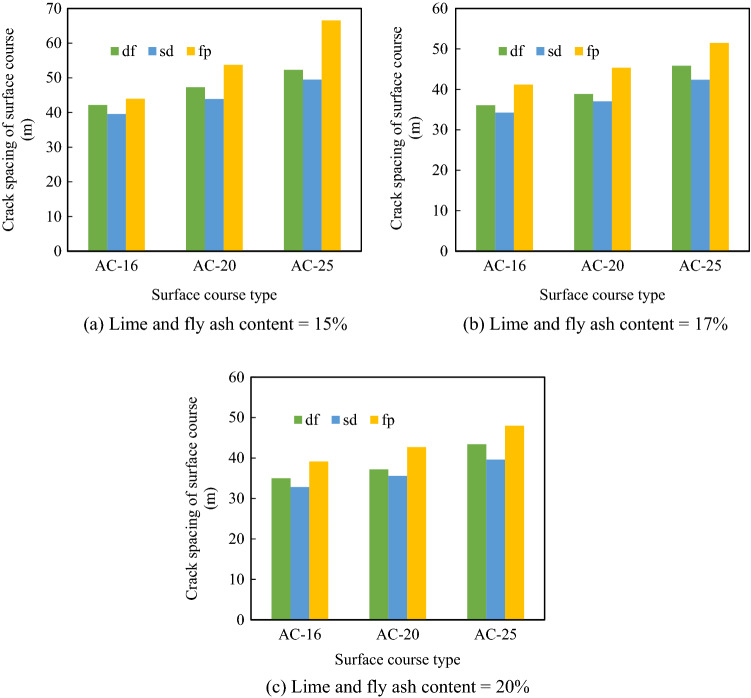
Crack spacings of the layer behind reflective cracks in different pavement structures.

When cracks in the base layer propagated to the surface layer, cracks in the surface layer were the superposition of cracks in the surface layer and reflective cracks. To calculate the transverse crack spacings of different composite structures, the results in Section “Transverse crack spacing in the pavement composite structure” were used. The temperature drop range was 48 °C. The composite structure with a length of 1 km was taken as the research object, and it was assumed that cracks in the inner surface layer per one km did not coincide with reflective cracks. The crack spacings of different pavement structures are presented in Fig. 20.

In the semi-rigid base asphalt pavement, after the appearance of reflective cracks, the transverse crack spacing on the surface layer ranged between 32.8 m and 66.5 m. Moreover, transverse crack spacing was related to the surface layer type. When the surface layer material was AC-16, the crack spacing was noticeably small. It happened because the smaller the particle size, the larger the temperature shrinkage coefficient and the more prominent the shrinkage and the more serious the transverse cracking. Transverse crack spacing was also related to the gradation type of the base layer. The crack spacings for the df-grade base and the fp-grade base were larger than that of the sd-grade base. Moreover, crack spacing gradually decreased with the increase in the content of lime and fly ash, especially for the sd-grade base and the df-grade base. Therefore, to control the transverse crack spacing of the surface layer after the appearance of reflective cracks, the particle size of the asphalt concrete in the lower layer needs to be appropriately increased, df or fp type gradation should be selected for the lime and fly ash-stabilized macadam base, and the dosage of lime and fly ash must be reduced.

### Recommendation for the best structural combination of the semi-rigid base asphalt pavement

The macadam base with the lime and fly ash content of 15% had the largest transverse cracking distance followed by the macadam base with the lime and fly ash content of 17% and the macadam base with the lime and fly ash content of 20%. Therefore, when the base has sufficient strength, the amount of lime and fly ash must be reduced to control cracking in the base layer. The sd-grade base had a small crack spacing; thus, sd gradation should be avoided for the lime and fly ash-stabilized macadam base. Moreover, the fp-grade base also had a small crack spacing, which should also be avoided during the design of asphalt pavements. The crack spacing of AC-25 was the largest, whereas that of AC-16 was the smallest. Therefore, the particle size of the asphalt mixture must be appropriately increased to control transverse cracking in the surface course. Hence, 15fp-AC25, 15fp-AC20, 15df-AC25, and 17fp-AC25 are the recommended semi-rigid base asphalt pavement structures to reduce transverse cracking.

## Conclusions

In this work, a calculation model of transverse crack spacing for a semi-rigid base asphalt pavement was established. The transverse crack spacings of different composite structures were calculated, and the influences of the shrinkage coefficient, the structural layer thickness, and the pavement tensile strength on transverse crack spacing were expounded. The transverse crack spacing of the pavement after the appearance of reflective was calculated. The following are the main conclusions from this study:

- The transverse crack spacing calculation model was established. It is worth noting that the surface course and base course of the pavement are assumed to be closely bonded, and the influence of interlayer slip is note considered.
- The lower lime and fly ash content and skeleton gap gradation can be adopted during the design of the base course. Meanwhile, the lower lime and fly ash content in the macadam base, the skeleton gap gradation and asphalt concrete with a larger nominal maximum aggregate size in the surface layer can be used during the design of surface layer.
- The transverse crack spacing of the semi-rigid base asphalt pavement could be increased by reducing the shrinkage coefficient, increasing the thicknesses of the surface course and the base course, and improving the tensile strength of the pavement.
- After the appearance of reflective cracks, the transverse crack spacing of the surface layer ranged between 32.8 m and 66.5 m. 15fp-AC25, 15fp-AC20, 15df-AC25, and 17fp-AC25 were found to be the best semi-rigid base asphalt pavement structures to reduce transverse cracking.
- Some limitations still exist in this research. It is assumed that the bonding between the surface layer and the base layer is continuous. However, the bonding is different in actual scenarios. Meanwhile, only numerical analysis was performed in this study without any model validation. Therefore, transverse cracking in pavement composite structures under different bonding conditions needs to be analyzed in the follow-up work. The experimental work will also be carried out in the next step to verify the model.

## Data Availability

All data included in this study are available from the corresponding authors and can be provided upon request as needed.

## References

[1] Hernando D, Val MAD (2013). A comprehensive overview on main distress mechanisms in composite pavements. Int. J. Pavement Res. Technol..

[2] Cheng PF, Zhou XP, Hou EC (2012). Experimental research about shrinkage of cement stabilized gravel base. Appl. Mech. Mater..

[3] Sharpe, G.W., Deen, R.C,, Southgate, H.F., Anderson, M. Pavement thickness design using low strength (pozzolanic) base and subbase materials.213–216 (1985).

[4] Feng PJ (2011). Contrast test for temperature shrinkage property of semi-rigid base materials. Transp. Stand..

[5] Wang L, Xie XG, Mei W (2012). Dry shrinkage characteristics of fly lime and cement stability aggregate materials of urban road. J. Dali. Marit. Univ..

[6] Rajbongshi P, Das A (2009). Estimation of temperature stress and low-temperature crack spacing in asphalt pavements. J. Transp. Eng..

[7] Prieto-Muñoz PA, Yin HM, Buttlar WG (2013). Two-dimensional stress analysis of low-temperature cracking in asphalt overlay/substrate systems. J. Mater. Civ. Eng..

[8] Yin HM (2010). Opening-mode cracking in asphalt pavements: crack initiation and saturation. Road Mater. Pavement Des..

[9] Timm, D. H. A phenomenological model to predict thermal crack spacing of asphalt pavement, Doctoral thesis, University of Minnesota, (2001)

[10] Timm DH, Voller VR (2003). Field validation and parametric study of a thermal crack spacing model. J. Assoc. Asphalt Paving Technol..

[11] Coetzee, N. F., Monismith, C. L. Analytical study of minimization of reflection cracking in asphalt concrete overlays by use of a rubber-asphalt interlayer. (1979).

[12] Bao CY, Liu Q, Xia YJ, Cui Y, Cao ZY, Qian YD, Liu MH, Mu CQ, Wang HL (2022). Influence of crack spacing/layer thickness value on reflection crack propagation mechanism under low temperatures. Front. Earth Sci..

[13] Ni, F.J., Li, Q. A prediction procedure of reflective cracking growth life for cement stabilized base asphalt pavement. In *6th RILEM International Conference on Cracking in Pavements*: 211 (2008).

[14] Gao YY (2019). Theoretical analysis of reflective cracking in asphalt pavement with semi-rigid base. Iran. J. Sci. Technol. Trans. Civ. Eng..

[15] Aliha MRM, Sarbijan MJ (2016). Effects of loading, geometry and material properties on fracture parameters of a pavement containing top-down and bottom-up cracks. Eng. Fract. Mech..

[16] Chen, B. Analysis of Mechanic Behavior of Steel and Concrete Composite Beams. Master's thesis, Hunan University, 2008.

[17] Wu, Q. Research on asphalt pavement structure combination in order to control transverse cracking rate. Master's thesis, Harbin Institute of Technology, (2016).

[18] Shi WM, Jiang ZS (2004). Research on the calculation method of temperature stress of cement treated crushed stone base and the method of controlling temperature shrinkage crack. J. Highw. Transp. Res. Dev..

[19] Zhang P, Li QF, Liu CH (2009). Prediction of shrinkage cracking and corresponding cracking prevention measure of the semi-rigid base layer. Int. J. Pavement Eng..

[20] Zhao, Z. H. Study on integrated design of pavement structure and material based on transverse crack spacing control. Master's thesis, Harbin Institute of Technology, (2018).

[21] Yang, W. D. Study on shrinkage properties of semi-rigid base material. Master's thesis, Chang’an University, (2004).

[22] Tian, L. Research on anti-cracking characteristics of frame-densed cement stabilized graded crushed stone. Doctoral thesis, Harbin Institute of Technology, (2010).

[23] Feng DC, Song Y (2007). Study of test and evaluation method on interfacial combining state of asphalt pavement. J. Harbin Inst. Technol..

[24] Shen AQ, Jiang QH (2004). Influencing factor and appraising on anti-cracking of asphalt mixture at low temperature. J. Chang’an Univ..

[25] Ma B, Wei YP, Wang L, Zhao CZ (2010). Analysis on flexural tensile characteristics of asphalt mixture in cold plateau region. J. Highw. Transp. Res. Dev..

[26] Dhakal N, Elseifi MA, Zhang Z (2016). Mitigation strategies for reflection cracking in rehabilitated pavements–a synthesis. Int. J. Pavement Res. Technol..

[27] Chen HX (2003). Analysis on fatigue and break of mix-material in half-steel pitch road surface. Commun. Stand..

[28] Chen, D. Research on fatigue performance of TLA modified asphalt mixture. Master's thesis, Changsha University of Science & Technology, (2008).

